# The Role of CT cholangiography in the Detection and Localisation of Suspected Bile Leakage Following Cholecystectomy

**DOI:** 10.4021/gr500e

**Published:** 2012-11-20

**Authors:** Michael Kirk, Elan Kaplan, Ruwangi Udayasiri, Val Usatoff

**Affiliations:** a Department of Surgery, Frankston Hospital, Victoria, Australia; b Department of Surgery, The Alfred Hospital, Victoria, Australia

**Keywords:** Cholecystectomy, CT cholangiography, Bile leak

## Abstract

**Background:**

Most bile duct injuries are not recognized at the time of initial surgery. Optimal treatment requires early recognition. CT IVC has become increasingly important in identifying bile leaks and their source after cholecystectomy. Our study aims to report the outcomes of using CT IVC post operatively and how accurately it can detect or localise bile leaks.

**Methods:**

From 2000 - 2009, twenty patients were managed for suspected bile leak post cholecystectomy within the Alfred Hospital. The study included a retrospective evaluation of the initial procedure, presenting symptoms, site of ductal injury, diagnostic procedures and therapeutic interventions. Results were analysed to determine success of the imaging procedure, and to correlate imaging diagnosis with results both diagnostically and clinically.

**Results:**

Twenty patients had a suspected bile leak, of which 3 were detected at the time of surgery. Seven patients had a CTIVC as their primary investigation. It identified bile leak in 6 and the anatomical site in 5. One had a leak excluded and was managed conservatively.

**Conclusions:**

CT Cholangiography is a feasible and low-risk tool for imaging of the biliary tract in suspected bile leaks post cholecystectomy. It is a valuable non-invasive investigation that may help avoid endoscopic retrograde Cholangiography or surgery.

## Introduction

The aim of this study is to evaluate the diagnostic potential and clinical utility of computed tomography (CT) performed after administration of cholangiographic contrast material in patients with a suspected bile leak after cholecystectomy. These patients underwent a variety of surgical and non-surgical interventions, and all experienced good outcomes. This study suggests that CT cholangiography is an accurate, non-invasive and safe technique to investigate the biliary system for leaks following cholecystectomies.

Laparoscopic technology, developed in the late 1980s has transformed the management of gallstone disease over the last two decades [1]. In current practice, laparoscopic cholecystectomy (LC) has succeeded open cholecystectomy (OC) in the operative management of gallbladder stone disease. Operative injury of the biliary tree is not a new complication of cholecystectomy but has become increasingly more visible during the emergence of the laparoscopic approach [2-8]. Most bile duct injuries are not recognized at the time of the initial surgery with current data reporting the frequency of bile duct injuries occurring in 0.3-0.9% of cases [9-13]. Optimal treatment of bile leak resulting from a common duct injury relies upon early recognition and planning of a therapeutic approach to avoid adverse outcomes such as severe peritonitis, sepsis and pain. The majority of bile leaks, however, are not major duct injuries [14].

Patients with bile leak complications commonly present early in the post-operative period with vague abdominal pain, persistent nausea and vomiting, discomfort and a low-grade fever [15-17]. These patients are usually unwell post-operatively with either contained loculated collections in the gallbladder fossa or around the liver, or frank diffuse biliary peritonitis. If a drain tube is present, high volume bile output can also occur. CT scans are sensitive in detecting intraperitoneal or pelvic free fluid, lymphoceles, haematomas or bile leaks. CT IVC has become increasingly important in identifying bile leaks and their source following cholecystectomy. This study aims to report the outcomes of using CT IVC post operatively and how accurately it can detect bile leaks prior to definitive management.

## Materials and Methods

### Patients

Between 2000 and 2009, 20 patients were managed for symptomatic bile leak post cholecystectomy within the Alfred network. The study includes a retrospective evaluation of the initial procedure, presenting symptoms of the bile duct lesion, site of ductal injury, diagnostic procedures and therapeutic interventions. Information concerning the initial procedure was obtained from multiple sources including operative reports and medical files and charts. Major presenting symptoms include pyrexia, abdominal pain and distension, persistent or higher than expected bile drainage from any drain tubes, nausea or vomiting. After clinical examination, all patients were investigated with routine haematological and biochemistry tests, including liver functions with added imaging modalities of abdominal ultrasound or CT in some cases. CT IVC involves CT scanning after intra-venous administration of the biliary contrast medium to generate three-dimensional images of the biliary tract. With the aid of this CT modality, duct anatomy is able to be visualised to view potential biliary filling defects or leakage. All CT IVC examinations performed within this study were transferred to a picture archiving and communication system (PACS) and interpreted by an experienced abdominal radiologist. Reports included a description of the presence or absence of a bile leak, and its location if present. Direct signs of a bile leak were related to extravasation of the contrast medium outside the biliary tree. Indirect signs were interpreted from either focal biliary dilatation or fluid collections.

Treatments varied from percutaneous drainage under ultrasound or CT guidance in those cases, in which a sizeable localized collection was apparent to laparoscopy and drain placement and/or endoscopic retrograde cholangiopancreatography (ERCP) to delineate biliary tree anatomy and to definitively treat with sphincterotomy or stenting.

### Data collection

A prospective database was reviewed to identify all patients treated at the Alfred Hospital between January 2000 and April 2009, which were complicated with a bile leak post cholecystectomy. Patient’s electronic and paper charts were retrospectively reviewed to analyse demographics, referring surgeon management, as well as peri-operative surgical management and outcomes.

Of the 20 patients involved, 18 were those that had their initial operation within the Alfred, one was referred post-operatively from Sandringham Hospital, and one post-operatively from Cabrini Hospital. CT IVC results were analysed retrospectively to determine technical success rate of the imaging procedure and to correlate imaging diagnosis with results of other diagnostic procedures and clinical follow-up. Bile leaks included all transections or partial lacerations of the common hepatic duct, common bile duct, or major segmental ducts at the porta hepatis. Minor leaks from the cystic duct or gallbladder bed were excluded. Only injuries and strictures incurred in association with surgery, irrespective of whether the operation was completed laparoscopic or converted to an open procedure, are included in the results.

## Results

The study included 14 women and 6 men with a mean age of 59 years (range 24 - 90). Patient demographics and management prior to referral are detailed in Table 1. Patients who had a symptomatic bile leak had a variety of surgical methods of cholecystectomy: LC in 13, OC in 3, LC converted to OC in 3, and LC converted to OC with transcystic common bile duct exploration in 1. Twelve patients (60%) underwent an intraoperative cholangiogram, and in 3 of those cases potential bile leakage was noted due to abnormalities with the imaging.The average amount of daily drain output was less than 150 mL/day in 10 patients and more than or equal to 150 mL/day in 8 patients. The mean interval between index surgery and detection of bile leak was 1.85 days (range 0 - 4 days). Documented suspicion of bile leakage clinically was most evident via increase bile drainage from surgical drains (n = 9) or acute abdominal pain (n = 11).

**Table 1:** Demographics and Initial Surgery

		Number (%)
Age (years)	Mean	59
Gender	Male	6 (30)
	Female	14 (70)
Place of Initial Operation	Study institution	18 (90)
	Other institution	2 (10)
Operation Type	Laparoscopic Cholecystectomy	13 (65)
	Open Cholecystectomy	3 (15)
	Laparoscopic converted to Open Cholecystectomy	3 (15)
	Laparoscopic converted to Open Cholecystectomy + CBD exploration	1 (5)
Recognition of injury by Laparoscopic surgeon		3 (15)

CT IVC was used in 7 of the cases, 5 post LC and 2 post OC, with identification of the leak in 6 of those procedures. The one case in which CT IVC was unable to identify a leak was post-operative open cholecystectomy. The actual site of bile leak was demonstrated in 57% of the cases using CT IVC as the primary imaging modality. Two cases noted bile leak from the gallbladder fossa, 2 from the cystic duct itself, and 2 cases unable to localize the leak.

Overall further laparotomy/laparoscopy was required in 5 patients, of whom 3 underwent a simple abdominal washout procedure, 1 underwent a washout with drain placement, and 1 that had a washout, oversew of bile leak and T tube placement. ERCP was required in 12 patients, and only in 4 of those who had a CT IVC looking for the leak post-operatively. There was no mortality associated with any of the procedures.

## Discussion

This study describes the use of CT IVC to investigate patients with a suspected bile leak post operatively in order to localise the leak, define the anatomy and identify ductal stones. There is an increasingly multidisciplinary approach to the diagnosis of bile leak following cholecystectomy that requires collaboration with surgeons, endoscopists and interventional radiologists. Alternative imaging modalities may not always be accessible [18-20]. Early diagnosis and management is vital in preventing further complications and the use of CT IVC for pre-operative workup is well recognised [21-22], however its usefulness for evaluation in the post-operative period is yet to be warranted.

CT IVC involves the intravenous use of the biliary contrast media and may induce anaphylaxis with the mortality rate recorded as 1 in 5000 [23-24], however this is less than the mortality rate in comparison to ERCP [25]. It also has limited value in those patients with an elevated serum bilirubin level [22]. For LC, the average incidence of bile duct injuries has been found at 0.36% [26].

Within this study CT IVC revealed bile leaks in 6 patients and excluded bile leaks in 14% of patients post gallbladder surgery. CT IVC helped to reduce the need for ERCP to only 12 of the patients with bile leakage and was directly used to support the ERCP in 4 of the patients by adding additional information.

### Conclusions

In conclusion, CT IVC is a feasible and relatively low-risk tool in the post-operative period for imaging of the biliary tract and may facilitate in avoiding the need for ERCP or further surgery. We recommended consideration of CT IVC as a non-invasive diagnostic tool in the detection and localisation of clinically suspected biliary duct leaks post cholecystectomy.
